# Plasma heat shock protein 27 is associated with coronary artery disease, abdominal aortic aneurysm and peripheral artery disease

**DOI:** 10.1186/2193-1801-3-635

**Published:** 2014-10-28

**Authors:** Cao Jin, Victoria L Phillips, Michael JA Williams, Andre M van Rij, Gregory T Jones

**Affiliations:** Department of Surgery, University of Otago, PO Box 913, Dunedin, New Zealand; Department of Medicine, University of Otago, PO Box 913, Dunedin, New Zealand

## Abstract

Low protein levels of Hsp27 have been reported in atherosclerotic plaques. In addition, human studies have indicated that circulating Hsp27 levels are lower in coronary artery disease patients compared with controls. It remains, however, unclear whether this applies to other forms of atherosclerotic disease.

Plasma Hsp27 from 280 subjects was examined by ELISA. The cohort included 80 coronary artery disease (CAD), 40 peripheral artery disease (PAD) and 80 abdominal aortic aneurysm (AAA) patients. Eighty elderly subjects, without any clinical history of vascular diseases, were used as a control group. Receiver operating curve (ROC) and logistic regression model analysis were performed to evaluate the potential value of Hsp27 as a circulating biomarker.

Patients with atherosclerotic vascular diseases had significantly lower levels of Hsp27 than control subjects (p < 0.001). Moreover, Hsp27 was significantly lower in CAD patients than other atherosclerotic vascular disease groups (p < 0.001). There was no difference in Hsp27 levels between the AAA and PAD groups. Using the ROC-generated optimal cut-off values for Hsp27, logistic regression modeling indicated that low plasma Hsp27 was independently associated with the presence of multiple forms of atherosclerotic disease.

In conclusion, circulating Hsp27 is significantly lower in patients with multiple forms of atherosclerotic arterial disease.

## Introduction

Atherosclerosis is a complex inflammatory disease process, which is the foundation of many vascular diseases (Libby 2002), including coronary artery disease (CAD), peripheral artery disease (PAD) (Sukhija et al. 2004, Sule et al. 2008) and abdominal aortic aneurysm (AAA) (Jones 2011). Although AAA has traditionally been considered to be an atherosclerotic phenotype, it has several distinctive features from that of occlusive atherosclerotic disease. These include differences in gender distribution, an apparently protective association with diabetes and a histopathology characterized by extensive medial atrophy and adventitial inflammation (Sakalihasan et al. 2005, Jones 2011).

Heat shock proteins are abnormally expressed in a variety of vascular diseases and are emerging as potential therapeutic targets (Christians et al. 2012, Henderson and Pockley 2012). Traditionally heat shock proteins are recognized as intracellular chaperones, yet both *in vitro* and *in vivo* studies have reported functional roles within both the extracellular domain and circulation (Laudanski et al. 2007, Shields et al. 2011). Regardless of their specific physiological roles, the presence of detectable protein levels within the circulation may make heat shock proteins potential diagnostic biomarkers. Heat shock protein 27 (Hsp2), also known as heat shock protein beta-1, is a member of the small heat shock protein family, which has been reported to have cyto-protective, anti-apoptotic, anti-oxidative and anti-inflammatory activity (Vidyasagar et al. 2012, Acunzo et al. 2014). An association between Hsp27 and atherosclerosis has been suggested, with several reports indicating that the expression of Hsp27 is inversely correlated with plaque burden in human carotid samples (Martin-Ventura et al. 2004, Martin-Ventura et al. 2006, Park et al. 2006, Lepedda et al. 2009). Moreover, in mouse models, overexpression of Hsp27 appeared to reduce aortic plaque burden and promote features of plaque stability (Rayner et al. 2009, Cuerrier et al. 2013, Seibert et al. 2013). Finally, cohort studies have suggested that circulating Hsp27 levels may represent a potential biomarker for human atherosclerosis, with reduced proteins levels being detected in patients with both carotid and coronary atherosclerosis (Martin-Ventura et al. 2004, Seibert et al. 2013). Nevertheless, it remains unclear whether lower levels of circulating Hsp27 represents a generalizable risk factor for all forms of atherosclerosis, such as that associated with smoking or lipoprotein profile (Jones et al. 2007).

This study therefore aimed to determine the relationship between plasma Hsp27 levels and several distinct forms of arterial vascular disease, including AAA, CAD and PAD, to further assess Hsp27 as a potential biomarker for atherosclerotic disease.

## Methods

### Study populations

Vascular disease patients, mainly of White ethnic origin (>97%), were recruited from the Otago region of New Zealand and compared with a healthy elderly control group from the same geographical region. All participants provided written informed consent, and the study was undertaken with the approval of the Regional Ethics Committee. A total of 280 patients were enrolled, in whom vascular disease status consisted of coronary artery disease (CAD, n =80), ischemic peripheral arterial disease (PAD, n =40) and abdominal aortic aneurysm (AAA, n =80). Elderly controls (Control, n = 80) were also recruited from the local community as previously described (Jones et al. 2013). In brief, inclusion criteria consisting of age >55 years, no previous history of ischemic heart disease (including angina pectoris), PAD, stroke (including transient ischemic attack) and being currently in good general health. Patients classified as having CAD all had angiographically proven coronary artery diameter stenosis of ≥50% in at least one vessel. The percentage diameter stenosis was visually estimated as the maximum reduction in the vessel diameter expressed as a percentage of the angiographically normal adjacent vessel diameter. Peripheral arterial disease was defined as a significant stenosis in multiple segments, including clinical symptoms such as claudication, rest pain, or tissue loss. The diagnosis of PAD was confirmed with a resting ankle-brachial index <0.7, pulse volume recordings, arteriography, and/or duplex arterial scan. All AAA patients had a maximum infra-renal aortic diameter >3 cm and were either undergoing surveillance (3-5 cm) or on active vascular review awaiting surgical repair (>5 cm). All other study participants (including controls) underwent an abdominal ultrasound examination to identify concurrent AAA, with incidental AAA being an exclusion criterion for the control group. The CAD with AAA group represented patients, undergoing coronary angiography, in whom an incidental observation of AAA (>3 cm) was made.

All study participants completed a questionnaire to ascertain demographic risk factors, including age, gender, history of hypertension and hyperlipidemia, and smoking habits (pack years). EDTA and heparin plasma samples were collected, centrifuged, separated into aliquots and frozen at -80oC within 30 minutes. Lipoprotein profiles and high sensitivity C-reactive (hsCRP) protein measurements were performed as previously described (Jones et al. 2009). An experienced clinical research technician assisted participants with questionnaire completion.

### Plasma HSP27 measurements

Hsp27 levels was measured in heparin plasma using a commercial ELISA kit according to the manufacturers instructions (Abcam, Cambridge, UK, ab108862). The assay detection range was 0.313- 80 ng/ml. The average coefficient of variation was <6%.

### Statistical analyses

All data is presented as the mean ± SD when normally distributed, otherwise the median (interquartile range, IQR) is shown. Statistical analyses were performed using an ANOVA test for age comparisons between groups and Mann–Whitney *U*-test for Hsp27, HDL-cholesterol and hsCRP comparisons between groups. The chi-square test was used to compare categorical data between each group. Receiver operating characteristic (ROC) curves were constructed to determine the optimal binary cut-off value of Hsp27 to differentiate vascular patients from elderly controls. This value was calculated using the maximum of the Youden index *J* = max [SEi + SPi-1], where SEi and SPi are the sensitivity and specificity over all possible threshold values. Differences were considered significant at p-values <0.05. A stepwise logistics regression analysis was then performed to establish if the binary cut-off value for Hsp27 was significantly associated with the presence of vascular disease, independent of other confounding variables including age, gender, smoking, diabetes, hypertension, dyslipidemia and hsCRP levels. Analyses were performed using Statview 5.01 (SAS Institute). The mROC (Unité de biostatistiques, CRLC Val d’Aurelle, V1.0) software package was used to perform receiver operator characteristic curves.

## Results

### Demographics

The characteristics of the study populations are summarized in Table 1. All groups were well-matched for age and gender. However, smoking pack years and history of hypertension and dyslipidemia were significantly higher in all four vascular disease groups compared with the vascular disease-free control group (p < 0.001). Diabetes was only significantly different between the control and CAD groups. C-reactive protein was higher in the CAD group than the control and AAA groups (p < 0.001).

**Table 1:** **Characteristics of study participants**

	Controls (n = 80)	AAA (n = 80)	PAD (n = 40)	CAD (n = 80)
Age (years)	72.7 ± 6.1	74.8 ± 8.1	72.1 ± 9.4	75.0 ± 8.6
Males (%)	66 (82.5)	63 (78.8)	32 (80.0)	67 (83.8)
BMI (kg/m^2^)	25.9 ± 3.5	27.1 ± 4.4	26.3 ± 4.1^†^	28.5 ± 4.5*
History IHD (%)	0 (0)	35 (43.8)^†^	12 (30.0)^†^	80 (100)
Smoking (pack years)	0 (0–12.3)	26.0 (10.0-42.6)*	23.8 (6.9-40.0)*	16.3 (0–33.1)*
Diabetes (%)	5 (6.3)	9 (11.3)	6 (15.0)	17 (21.3)*
Hypertension (%)	25 (31.3)	48 (60.0)*	26 (65.0)*	64 (80.0)*
Dyslipidemia (%)	25 (31.3)	43 (53.8)*	28 (70.0)*	60 (75.0)*
hs-CRP, mg/L	1.9 (1.1-4.7)	1.9 (0.9-5.1)^†^	2.2 (1.3-5.7)	4.5 (2.3-4.9)*
HDL-C mmol/L	1.22 (1.05-1.49)	1.11 (0.93-1.31)*	1.16 (0.96-1.53)	1.14 (0.93-1.31)
Hsp27, ng/ml	2.68 (1.78-4.42)	1.82 (0.91-3.37)*^†^	1.93 (1.01-3.05)*^†^	1.23 (0.84-2.12)*

Age and BMI are shown as mean ± SD; Hsp27 and smoking (pack years) are shown as median (IQR); The categorical data is presented as number (percentage). *p < 0.001 versus control group; ^†^p < 0.001 versus CAD group.

Body mass index (BMI), Ischemic heart disease (IHD), plasma high sensitivity C-reactive protein (hs-CRP), High-density lipoprotein cholesterol (HDL-C), Heat shock protein-27 (Hsp27).

### Plasma Hsp27 levels

Plasma Hsp27 was significantly lower in all of the vascular disease groups compared to the control group (Table 1). CAD patients had significantly lower Hsp27 than the other vascular disease groups (p < 0.0001). However, there was no significant difference in Hsp27 levels between the PAD and AAA groups. By univariate (inter and intra-group) analysis, the levels of Hsp27 were not significantly influenced by other cardiovascular risk factors, including age, gender, smoking (pack years), diabetes, dyslipidemia, hypertension and plasma hsCRP.

ROC curves were plotted to identify the optimal discrimination value for Hsp27 as a binary threshold to distinguish the various disease groups from controls. The greatest area under the ROC curve (AUROC) was achieved by an Hsp27 value of 2.24 ng/mL when comparing CAD patients with elderly controls (Table 2), with an AUROC value of 0.75 (95% CI: 0.67-0.82).

**Table 2:** **Comparison of Hsp 27 ROC curves in different arterial disease groups**

Patient group	Cut-off value (ng/ml)	Sensitivity (%)	Specificity (%)	AUROC	AUROC 95% CI
CAD (n = 80)	2.24	0.69	0.67	0.75	0.67-0.82
PAD (n = 40)	2.69	0.59	0.58	0.62	0.51-0.71
AAA (n = 80)	2.75	0.58	0.56	0.60	0.50-0.68
All arterial disease patients (n = 200)	2.63	0.61	0.60	0.65	0.58-0.71

Optimal cut-off values were calculated using the Youden index. Area under the receiver operating characteristic curve (AUROC).

Using the optimal ROC cut-offs for the respective vascular diseases, Hsp27 was shown to be significantly associated with CAD, PAD, AAA and the combined atherosclerotic vascular diseases group. These remained significant following adjustment for known cardiovascular risk factors, including age, gender, smoking pack years, diabetes, dyslipidemia, hypertension and hsCRP (Table 3).

**Table 3:** **Logistic regression for plasma Hsp27**

Group/binary threshold	Univariate odds ratio			Adjusted* odds ratio		
	Odds ratio	95% CI	P value	Odds ratio	95% CI	P value
All CAD (n = 80), Hsp27 > 2.24	0.17	0.08-0.34	0.0001	0.21	0.10-0.42	0.0001
PAD (n = 40), Hsp27 > 2.69	0.36	0.16-0.81	0.013	0.25	0.09-0.71	0.009
AAA (n = 80), Hsp27 > 2.75	0.43	0.22-0.82	0.011	0.29	0.12-0.69	0.005
All disease patients (n = 200), Hsp27 > 2.63	0.32	0.18-0.55	0.001	0.21	0.10-0.42	0.001
PAD & AAA (n = 120), Hsp27 > 2.63	0.40	0.22-0.72	0.002	0.26†	0.17-0.56	0.006

Logistic regression analysis was used to calculate the odds ratios for having plasma Hsp27 levels above binary thresholds. The reference population was the elderly control group. The adjusted model 1* included age, gender, history of hypertension, dyslipidemia, diabetes, smoking (pack years) and high sensitivity C-reactive protein. Adjusted model 2† included all factors in model 1 plus history of CAD.

Potential associations between plasma Hsp27 and severity of atherosclerotic disease burden was also assessed. In CAD patients the extent of (single, double or triple vessel) disease, showed a trend (p = 0.1) towards decreased Hsp27 in patients with triple versus single vessel disease was observed (median Hsp27 levels of 1.35, 1.19 and 1.08 ng/mL in 1, 2 and 3 vessel disease respectively). Plasma Hsp27 was not correlated with AAA size (rho = 0.05, p = 0.57) or severity of PAD as assessed by ankle brachial pressure index (rho = −0.15, p = 0.38). In all vascular disease patients, no association was observed between the number of concurrent diseases and plasma Hsp27 (data not shown). Plasma Hsp27 did not significantly correlate with circulating levels of hsCRP or HDL-choelsterol (data not shown).

In order to determine if the intermediate levels of Hsp27 in the PAD and AAA groups were due to the presence of sub-groups with concurrent CAD, these groups were further divided into those with or without a history of CAD. The presence of concurrent CAD did not result in any statistical difference in either median HSP27 (1.88 ng/mL versus 1.86 ng/mL in those with and without CAD respectively) or the proportion below the optimal binary threshold of 2.69 ng/mL (69.6% versus 70.3%, with and without respectively, p = 0.87). Logistic regression analysis also suggested that the lower levels of Hsp27 observed in AAA and PAD patients remained significantly different from controls after adjustment for the presence of concurrent CAD (Table 3).

## Discussion

In this study we demonstrated for the first time that plasma levels of Hsp27 are a potential biomarker for several distinct atherosclerotic vascular diseases.

Although it has been previously reported that circulating Hsp27 is significantly lower in both CAD and carotid stenosis patients compared with control subjects (Martin-Ventura et al. 2004, Seibert et al. 2013), it was not clear if such an association existed in other forms of atherosclerotic vascular disease, such as PAD and AAA. In this study Hsp27 was found to be lower in CAD patients consistent with previous reports, however, we also demonstrated that Hsp27 was significantly lower in AAA and PAD patients compared with a group of elderly controls (with no previous history of cardiovascular diseases). While plasma Hsp27 appeared to be reduced in all vascular disease groups, the CAD group had significantly lower levels than the other disease groups. However, because of the high degree of concurrent CAD within both the AAA and PAD groups, the low levels of Hsp27 in these two groups could conceivably be due to a CAD specific Hsp27 effect. Indeed, the presence of concurrent disease is a significant limitation of this current study design. Although the presence of AAA was excluded from other vascular disease groups, we cannot fully discount the interactive effects of concurrent vascular disease between the case groups. Nevertheless, sub-analysis suggested that levels of Hsp27 were similar in AAA and PAD patients regardless of the presence or absence of concurrent CAD, while logistic regression appeared to indicate that the significantly lower plasma Hsp27 levels in AAA and PAD patients (compared with elderly controls) are independent of concurrent CAD (Table 3). Our analysis therefore appears to suggest that lower circulating levels of Hsp27 in arterial disease patients is not specific to CAD, but rather is a relatively ubiquitous finding amongst atherosclerotic vascular disease phenotypes.

To further assess the utility of Hsp27 as a biomarker for atherosclerotic diseases, we performed ROC analysis to determine the optimal binary cut-off values to distinguish between atherosclerotic cases and controls. The greatest discrimination was observed when comparing CAD patients with elderly controls. Using a binary cut-off value we were able to demonstrate a significant univariate association with all forms of vascular disease. This remained as an independent protective association following adjustment for known risk factors. While these observations are encouraging, it should be noted that this study is limited by its retrospective design and clearly further prospective cohort studies are required to assess the clinical utility of plasma Hsp27 as a predictor of (early asymptomatic) atherosclerotic disease.

Nevertheless, our current observation of reduced plasma Hsp27 in patients with vascular disease appears to be consistent with previous reports suggesting reduced Hsp27 levels within atherosclerotic plaques (Martin-Ventura et al. 2004, Martin-Ventura et al. 2006). Functionally, hsp27 is a known potent anti-apoptotic protein (Ferns et al. 2006, Ghayour-Mobarhan et al. 2012, Vidyasagar et al. 2012). Since apoptosis of vascular smooth muscle is involved in the weakening of the fibrous cap and therefore plaque rupture, the reduced expression of protective Hsp27 may favor the formation of unstable plaque (Ghayour-Mobarhan et al. 2012, Vidyasagar et al. 2012, Raizman et al. 2013). In addition, Hsp27 has been implicated as a regulator of tissue inflammation, a pivotal process in atherosclerosis (Libby 2002). It has been suggested that Hsp27 can lower the levels of the inflammatory cytokine IL-1β in macrophages and enhance extracellular levels of the anti-inflammatory cytokine interleukin-10 (IL-10) (De et al. 2000, Rayner et al. 2008). Extracellular Hsp27 can also attenuate foam cell formation and atherogenesis by down-regulating NF-κB signaling in macrophages (Raizman et al. 2013, Salari et al. 2013). However, it should be noted that a post-hoc analysis of our data did not find any significant correlation between circulating Hsp27 and the inflammatory related markers hsCRP or HDL-cholesterol.

Given the consistent reductions in Hsp27 observed across arterial disease groups it is perhaps surprising that we did not observe any strong associations between plasma Hsp27 and either the extent of coronary artery disease or burden of concurrent disease. Nevertheless, in this study we did observe a non-significant trend towards lower plasma Hsp27 with increasing severity of CAD, which appears to be consistent with a study by Pourghadamyari and colleagues, in which they reported a significant positive correlation between serum Hsp27 antibodies and severity of CAD (Pourghadamyari et al. 2011).

In conclusion, the findings of this study are consistent with previous reports indicating that low plasma Hsp27 levels are associated coronary artery disease. In addition, we demonstrated that other forms of arterial disease, including both occlusive (PAD) and aneurysmal (AAA) phenotypes, also have lower levels than healthy elderly controls. Plasma Hsp27 remained significantly inversely associated with arterial disease after adjusting for confounding risk factors including age, gender, history of hypertension, dyslipidemia, diabetes, smoking and hsCRP. Taken together, these observations appear to suggest that future investigations are warranted in order to determine if Hsp27 is a useful circulating biomarker for determining both susceptibility and severity of atherosclerotic disease burden within a population.
